# The Use of X-Rays in the Diagnosis of Pulmonary Tuberculosis

**Published:** 1913-03-08

**Authors:** 


### The Use of X-Rays in the Diagnosis of Pulmonary Tuberculosis.

Under the presidency of Dr. Reginald Morton, the Electro-Therapeutical Section of the Royal Society of Medicine on Friday, 21st ult., discussed this question, which eventually was adjourned until the next meeting.

Sir Richard Douglas Powell said that no one appreciated more highly than he the importance of $x$-ray work, and the improved technique which had brought it to its present stage of usefulness. The object of $x$-ray photographs in relation to phthisis was to concentrate into one view the physical conditions present in the lungs, and thus to refute, correct, or confirm the findings by ordinary physical examination. Dealing with Dr. Jordan's published work, he did not think that one could interpret infiltration of the bronchial glands, or even streaks of tissue leading from them, as necessarily tuberculous; and he would require pathological justification of the view so based. The bronchial glands he regarded as the dustbins of the bronchial tract, and said they collected unconsidered trifles which might gather in the bronchial tubes and get into the lymph streams. Therefore, disturbance of the bronchial regions need not be tubercular. Dr. Jordan had found, in *post-mortems* on supposedly healthy persons who had died of accident, that tuberculous disease was present in 33 per cent.

The bronchial glands were also the lethal chambers of tubercle bacilli. His difficulty was in understanding how the lung should be secondarily invaded by the spreading of the tuberculous poison from the glands radiating out towards the periphery of the lungs. The radiograms he had seen were not convincing to him as indicating the commencement of the tuberculosis at the root of the lung. He felt sure that with the beautiful instalments of apparatus at hospitals mistakes connected with skiagrams and their interpretation would be eliminated; and periodic pictures of cases at sanatoria would mark progress more readily than much written description. But there was the danger that the fibrosis of healing phthisis might be regarded, owing to the shadow, as an extension of the disease. He believed the rays would prove of considerable value in the definition of cavities, particularly with the view of surgically treating them, the recognition of empyæmata, and the outlines of pneumothorax. But for recognising cases of early phthisis the method would probably be of value only in a few selected cases. It was valuable in all cases in the sense that auscultation and percussion were valuable. For scientific progress, for demonstration, and for giving precision of teaching at schools, $x$-ray apparatus was of the highest value.

Dr. David Lees said that seven years ago, as a result of joint but independent examination of a number of cases by ordinary methods by himself and by radiography at the hands of Dr. Simmons, they concluded that $x$-rays would not definitely show areas of apical dulness, which to his, Dr. Lees', percussion were quite evident, and that hence the old methods of examination were more trustworthy. But he anticipated then that improvements in technique would in time alter that conclusion, and such had proved to be the case, as shown by a series of cases examined by him on the usual lines, and by Dr. Orton by $x$-rays. Still, he believed that the value of careful and accurate percussion in the detection of incipient pulmonary tuberculosis was much under-estimated; and it would not be sufficiently appreciated until physicians gave up the habit of examining patients in the erect posture. In every one of the series seen by himself and Dr. Orton, where percussion gave reason to believe incipient pulmonary tubercle existed, Dr. Orton found very definite shadow signs of the disease. But it must be remembered that some of the shadows cast might be those of damaged glands and thickened bronchial tubes as a result of past activity; hence the most expert radiographers should be employed, and every care given to interpretation. The rays had demonstrated the occurrence of a nervous inhibition of the diaphragm in some cases, so that no persuasion would result in the taking of a deep breath. His general conclusion was that $x$-rays were likely to be of great service in the detection of early pulmonary tuberculosis, but not of necessary service, for the same facts were determinable by careful percussion.

Dr. Jordan confirmed his previous observations as to the significance of peribronchial mottling, whether the apices were involved or not, and narrated a case in which he localised five areas of disease in a girl's lung, and lettered them, and when shortly afterwards she met with a fatal accident and was examined *post-mortem*, the areas were exactly confirmed. The discussion will be continued.

